# 

**Published:** 1851-05

**Authors:** 


### CONTENTS:

### PART I.—ORIGINAL COMMUNICATIONS.

ART. 1.—Clinical Lectures and Cases, by Prof. Davis, - - - - - - p. 1
" 2.—History of Medical Education and Institutions—Reviewing in general, by Prof. Davis, - - - - - - - - - - - - - 17
" 3.—Compression of the Abdominal Aorta, by Ira E. Oatman, M.D. - - - - 33
" 4.—Cases of Puerperal Convulsions, by Joel E. Hendrick, M.D. - - - - 35
" 5.—Report of Committee on Mortality and Hygiene, made to Chicago Medical Society, April 7th, 1851, by Prof. Herrick. - - - - - - - - 38
" 6—Observations on the use of the Obstetrical Extractor, by Prof. Evans, - 40
" 7.—Case of supposed violence or poisoning, by E. C. Banks, M.D. - - 46
" 8.—Treatment of Puerperal Convulsions, by Dr. R. Hull, - - - - - 48

### PART II.—REVIEWS AND NOTICES OF NEW WORKS.

ART. 1.—Review of Chemistry for Students, by John G. Murphy, M.D. - - - 49
" 2.—Dissertation on Medical Delusions, by Worthington Hooker, M.D. - - 50
" 3.—Reports of Lunatic Asylums, - - - - - - - - 55
" 4.—Proceedings of the Medical Association of the State of Alabama, - - 57

### PART III.—SELECTIONS.

ART. 1.—Hot Water in Sprains, - - - - - - 59
" 2.—Chiniodine in Intermittent Fever, - - - - - 65
" 3.—Deaths of distinguished Physicians, - - - - - - 68
" 4.—Accidental employment of Chloroform in Erysipelas, - - - 69
" 5.—Eruptions of the skin during Pregnancy, - - - - - 70
" 6.—Morphine in Strangulated Hernia, - - - - - - 71
" 7.—The Catoptric Test for Cataract, - - - - - - 73
" 8.—Local application of Chloroform in Sciatica, etc., - - - - 7[illegible]
" 9.—Partial Qualitative Analysis of the Tomato, - - - - - 78

### PART IV.—EDITORIAL.

ART. 1.—The Illinois State Medical Society, - - - - - - 81
" 2.—Perplexities, - - - - - - - - - 82
3.—Statistics of Medical Schools, - - - - - - - 84
" 4.—Miscellaneous Medical Intelligence, - - - - - - 85
Obituary, - - - - - - - - - - - - 88

